# Statistical learning prioritizes abstract over item-specific representations

**DOI:** 10.3758/s13423-025-02757-8

**Published:** 2025-09-03

**Authors:** Mei Zhou, Shelley Xiuli Tong

**Affiliations:** Human Communication, Learning, and Development, Faculty of Education, The University of Hong Kong, Hong Kong, China

**Keywords:** Statistical learning, Working memory, Internal representation, Abstract knowledge, Item-specific encoding

## Abstract

Statistical learning optimizes limited working memory by abstracting probabilistic associations among specific items. However, the cognitive mechanisms responsible for the working memory representation of abstract and item-specific information remain unclear. This study developed a learning-memory representation paradigm and tested three participant groups across three conditions: control (Experiment 1), item-specific encoding (Experiment 2), and abstract encoding (Experiment 3). All groups were first shown picture–artificial-character pairs that contained abstract semantic categories at high (100%), moderate (66.7%), and low (33.3%) probability levels and item-specific information (16.7%). Participants then completed an online visual search task that simultaneously assessed statistical learning and memory representation by examining how abstract or item-specific distractors influenced their speed for searching artificial characters. In the control condition, participants spent more time searching abstract than item-specific distractors across all probability levels, indicating abstract prioritization. In the item-specific condition, abstract prioritization was absent. In the abstract condition, enhanced prioritization of abstract information was observed for moderate and low, but not high, probability items. These findings suggest that statistical learning is central to the abstraction process, with input probabilities and encoding strategies jointly shaping the formation of abstract and item-specific representations. This process depends on a flexible working memory system that dynamically adjusts prioritization, particularly when inputs are uncertain.

**Supplementary Information:**

The online version contains supplementary material available at 10.3758/s13423-025-02757-8.

Statistical learning enables humans to automatically abstract patterns of environmental inputs that comprise a network of specific stimuli with varying connection strengths (Qi & Zevin, 2024; Saffran et al., 1996; Thiessen, 2017). Deeply rooted in the brain, this process optimally represents and organizes diverse sensory inputs to navigate the challenge of limited memory resources (de Sousa et al., 2021; Sun et al., 2023; Umemoto et al., 2010). Neuroimaging studies have shown that statistical learning can hinder the encoding of episodic details for reliably predictive (100%) inputs when individuals make efficient predictions (e.g., Sherman & Turk-Browne, 2020). Furthermore, memory representation is subject to individuals’ encoding strategies (e.g., Denis et al., 2023), or how they represent sensory inputs at the initial stage of the memory process (Hertzog et al., 2008). However, during online statistical learning, how different types of information are represented in working memory remains unexplored. This is partly due to the lack of an experimental paradigm that can track internal memory representations of probabilistic inputs while simultaneously manipulating encoding strategies. Using a novel learning-memory representation paradigm, this study investigated memory prioritization of abstract and item-specific representations during statistical learning with varying probabilities in different encoding strategies.

Item-specific and abstract information are prevalent in statistical learning stimuli, with item-specific details representing low-level attributes like appearance and concrete definitions, and abstract concepts linking various inputs at a higher level, such as semantic categories (e.g., Hunt & Einstein, 1981; McClelland et al., 1995; Schapiro et al., 2017). The complementary hippocampal operations for representing statistics and episodes (C-HORSE) model proposes that item-specific (episodic) and abstract (statistically learned) memory representations are generated through distinct yet interconnected hippocampal pathways (Singh et al., 2022; Zhou et al., 2023). While C-HORSE has successfully simulated human offline categorization of similar inputs (e.g., Sučević & Schapiro, 2023), it does not clarify how real-time memory representations of abstract and item-specific information are generated during online statistical learning, nor does it specify the underlying cognitive mechanisms involved.

Previous studies have suggested that individuals selectively prioritize a certain type of information when multiple types are present (e.g., Greve et al., 2017; Sherman & Turk-Browne, 2020). However, conflicting findings have emerged regarding which type of representation to prioritize. Structured inputs have been shown to facilitate the formation of abstract patterns and concepts at the expense of retaining specific details (Schapiro et al., 2017). For example, Sherman and colleagues (Sherman et al., 2022; Sherman & Turk-Browne, 2020) presented participants with pairs of natural scenery images, where the category of the first image consistently (i.e., 100%; predictive condition) or randomly (i.e., nonpredictive condition) predicted the category of the second image. They found that participants remembered predictive scenes less accurately than non-predictive ones, demonstrating that acquiring abstract category patterns impaired the episodic memorization of specific scenes (Sherman et al., 2022; Sherman & Turk-Browne, 2020). Conversely, item-specific memories can be prioritized, particularly when inputs contain unexpected elements (Bein et al., 2021; Greve et al., 2017). For example, in a study where participants learned the association between scene categories and words, a decrease in association probability from 100% to 83.3% resulted in enhanced memories of specific words as a product of error-driven learning (Greve et al., 2017).

Thus, these previous studies (e.g., Greve et al., 2017; Sherman et al., 2022; Sherman & Turk-Browne, 2020) suggest that the prioritization of abstract versus item-specific information depends on statistical learning contexts, particularly in relation to probability. However, as they focus on one type of representation within a high probability context only, none of them address whether and, if so, how abstract and item-specific representations are simultaneously adapted to different contexts within a single statistical learning experiment. To address this issue, the current study systematically investigated the co-existence of abstract and item-specific representations across high, moderate, and low levels.

Indeed, probability (i.e., an event’s likelihood or the co-occurrence of multiple items) varies in environmental inputs, such as written language. For example, in Chinese orthography, over 80% of characters (e.g., 跑/pao3/, *to run*) contain a semantic radical (e.g., 足, *foot*) indicating categorical meaning (e.g., *foot*-related concept) and a phonetic radical (e.g., 包/bao1/) providing a pronunciation cue (e.g., /ao/-rhyme; Shu et al., 2003). According to a character corpus analysis, the probability that a semantic radical represents its character’s semantic category ranges from 94% to 8% (Wang & Zhang, 2016). By simulating these semantic regularities using artificial logographic characters, a previous study showed a lower recognition accuracy for high-probability picture–artificial-character pairs where the semantic radical indicated its semantic category with 100% probability, compared with lower probability (60%) items for this association (Tong et al., 2020). This finding suggests that high-probability inputs, which are deterministic and rely more on abstract rules (Habiby Alaoui et al., 2021; Lee et al., 2022), tend to prioritize abstract information. Conversely, lower probability inputs, which are probabilistic and require precise evaluations of outcomes for a specific exemplar (Okazawa & Kiani, 2023), may enhance representations of item-specific information. Thus, we hypothesize that as probability decreased, internal representation would prioritize item-specific more than abstract information.

Another important factor that influences working memory representations during online statistical learning is the encoding strategy prompted by task instructions. As the initial stage of working memory (Melton, 1963), encoding determines how perceived information is maintained and retrieved. Working memory representations can vary by encoding strategies even when processing identical probabilistic inputs (Bo et al., 2011; Bo & Seidler, 2009). For example, in an implicit sequence learning task, working memory positively correlated with general skill acquisition rather than specific structure details (Bo et al., 2011). Conversely, when the same task became explicit (i.e., participants were informed of the existence of fixed sequences before learning the same structures), working memory associated with item-specific details (i.e., chunk length; Bo & Seidler, 2009). These conflicting findings may result from differences in encoding strategies (i.e., item-specific vs. abstract encoding), or the processing of stimulus details in the explicit but not implicit task. However, the impact of other encoding strategies, such as an abstract one that emphasizes overarching input patterns, remains unclear. Therefore, the current study systematically investigated how an item-specific and an abstract encoding strategy each influenced the internal representation prioritization processes.

Furthermore, our investigation of encoding strategy is motivated by two contrasting hypotheses regarding how different probability levels are processed. The probability-invariant hypothesis, based on Hebbian learning theory, suggests that memory strength depends solely on external statistics, and predicts a uniform encoding process across different probability levels, regardless of individual strategies prompted by task instructions (e.g., Hebb, 2005). In contrast, the probability-variant hypothesis, derived from the multicomponent view of statistical learning, proposes that working memory mechanisms differ across probability levels. It suggests that specific encoding strategies are tailored to particular learning contexts (e.g., Conway, 2020; Lee et al., 2022). Previous studies in passive-learning contexts could not distinguish between these hypotheses, as both accounted for better learning or memory of higher probability inputs (e.g., Endress, 2024; Tong et al., 2023).

However, by examining the effect of encoding strategy on the internal representations at each probability level, the present study tests these two hypotheses. If probability-invariant hypothesis is correct, a consistent effect of the prompted encoding strategy should be observed across all probability levels. Alternatively, if the probability-variant hypothesis holds, an interaction between probability and encoding strategy is expected. Specifically, high-probability items, which favor abstract information, will benefit more from an abstract encoding strategy, enhancing their abstract representations. In contrast, low-probability items, conductive to item-specific encoding, will be influenced more by an item-specific strategy, strengthening their detailed representations.

To test these hypotheses, a novel experimental paradigm that combines the working-memory–guided-attention paradigm (Fu et al., 2021) with the artificial orthography learning paradigm (Tong et al., 2020) was developed to track online working memory representations during statistical learning. The working-memory–guided-attention paradigm directly examines to what extent a specific piece of information is represented in working memory. The core logic of this paradigm is that features retained in working memory automatically direct participants’ attention to matched features during a subsequent visual search phase (e.g., Fu et al., 2021). By combining this paradigm with the artificial orthography learning paradigm (Tong et al., 2020), we systematically manipulated the association between artificial characters with semantic categories and specific meanings. These artificial characters comprised semantic radicals (i.e.,  ,  ,  ,  ,  ,  ) that associated with a semantic category at a high (100%), moderate (66.7%), or low probability (33.3%), and control radicals (i.e.,  ,  ,  ,  ,  ,  ) that randomly associated (16.7%) with the semantic categories. However, the semantic and control radicals were associated with specific meanings within the same category at 16.7% and 100% probabilities, respectively (Fig. 1D). This manipulation resulted in semantic radicals informing abstract information and control radicals informing item-specific information. If during the visual search phase participants respond slower under semantic-match compared with control-match distractors, a stronger working memory representation of abstract compared with item-specific information is indicated, which is called internal representation prioritization. This prioritization also reflects a statistical learning effect, given that semantic radicals have a stronger association with their corresponding semantic categories compared to the control radicals. Furthermore, we used a memory task that oriented different groups of participants to focus on either the specific item (e.g., bread) within each picture (i.e., item-specific encoding in Experiment 2) or the semantic category (e.g., food) of each picture (i.e., abstract encoding in Experiment 3), which was contrasted with a control group involving no memory task (Experiment 1).

**Fig. 1:**
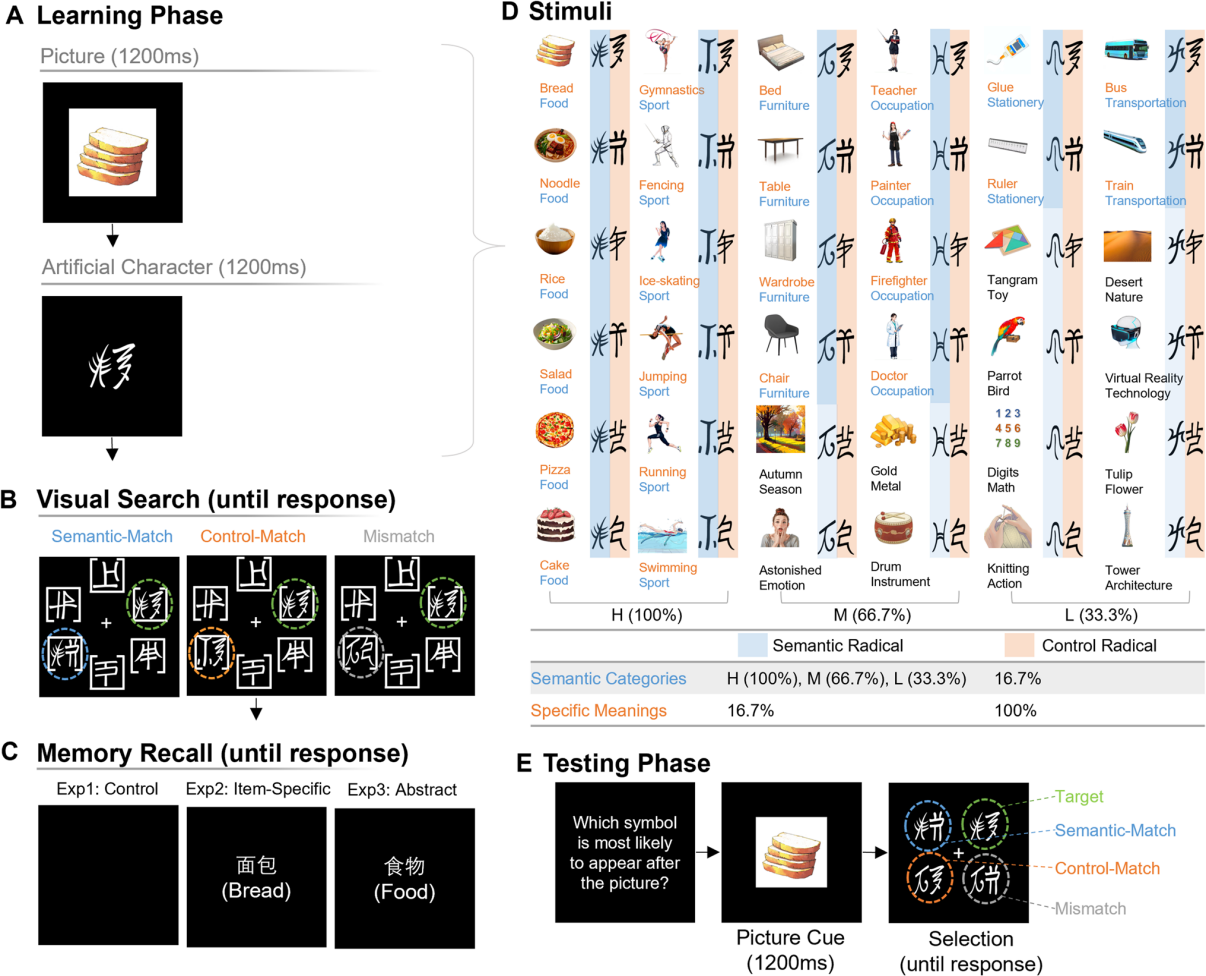
Schematic illustration of the experimental design, stimuli, and procedure. The experiment, embedded with high (H), moderate (M), and low (L) probabilities of picture and artificial character associations (**D**), with each picture–character pair presented to participants sequentially (**A**), followed by a visual search test (**B**) and a memory recall test (**C**). After the entire learning phase, participants completed a testing phase (**E**) by selecting from four choices (i.e., target, semantic-match, control-match, and mismatch) the character most likely to appear after a given picture. (Color figure online)

In sum, this study addressed two research questions: How does probability influence internal representations of abstract and item-specific information during online statistical learning (Experiment 1)?; and to what extent does the item-specific encoding strategy (Experiment 2) and the abstract encoding strategy (Experiment 3) influence the internal representations of different types of abstract and item-specific information across probability levels? Based on previous diverse findings (e.g., Bein et al., 2021; Sherman et al., 2022), two specific hypotheses were tested. First, abstract information was prioritized during statistical learning for high-probability but not low-probability items. Second, encoding strategies influenced this probability effect such that item-specific encoding boosted item-specific information for low-probability items, whereas abstract encoding facilitated abstract information for high probability items.

## Experiment 1

### Method

#### Participants

Participants were 97 university students (*M*_age_ = 22.70 ± 2.78 years; 73 women, 24 men) with normal or corrected-to-normal vision acuity and no neurodevelopmental disorders or brain damage. They provided written informed consent and were compensated 50 HKD (6.40 USD). This study was approved by the authors’ university’s Human Research Ethics Committee.

#### Stimuli

The core stimuli were six categorical sets of six (i.e., 36) artificial left-right-structured logographic characters (e.g.,  ) paired with corresponding pictures indicating their meanings (Fig. 1D). Each artificial character was created using a semantic radical (e.g.,  ) and a control radical (e.g.,  ) based on Dongba or Geba characters of Naxi scripts from western China used in the 17th century (Xu, 2023), and were unfamiliar to the participants. Both the position and the identity of the semantic radical were counterbalanced, with participants randomly assigned to one of four conditions: Dongba or Geba characters as semantic radicals on the left or right.

The artificial characters were classified into high (100%), moderate (66.7%), and low (33.3%) probability categories, with each category of artificial characters representing related meanings. The six artificial characters in each themed category (e.g., food) shared the same semantic radical (e.g.,  ) and used different control radicals (e.g.,  ,  ) to represent specific items in this category (e.g.,   ). The control radicals were consistent across six themed categories (i.e., food, sport, furniture, occupation, stationery, and transportation). Thus, each semantic and control radical exhibited the same frequency (i.e., six). As shown in Fig. 1D, both high-probability (100%) categories contained six artificial characters representing related meanings (food: *bread, rice*, *noodle*, *salad, pizza*, and *cake*; sport: *gymnastics*, *fencing*, *ice skating*, *jumping*, *running*, and *swimming*). Both moderate probability (66.7%) categories included four artificial characters representing related meanings (furniture: *bed*, *table*, *wardrobe*, *chair*; occupation: *teacher*, *painter*, *firefighter*, *doctor*). Both low-probability (33.3%) categories comprised two artificial characters representing related meanings (stationery: *glue* and *ruler*; transportation: *bus* and *train*).

The corresponding pictures that provided meaning to the artificial characters adhered to three principles: 1) related items within each category were semantically similar (e.g., *pizza*, *salad* [food]; *teacher*, *painter* [occupation]); 2) unrelated items within each category were semantically dissimilar (e.g., *autumn*, *astonished*; *gold*, *drum*); 3) no items across categories were semantically similar (e.g., *noodle*, *fencing*, *table*; *drum*, *knitting*, *tower*). Semantic similarity was evaluated by 24 adults who rated *the extent to which two words relate to each other in terms of meaning* using a 7-point Likert scale (1 =* not related, *7 = *very related*; see details in Supplemental Fig. S2).

#### Procedure

Before the formal experiment, participants named all pictures used in the experiment and received immediate feedback.

The experiment comprised a learning phase and a testing phase (Fig. 1). In the learning phase, participants were sequentially shown a picture, its corresponding artificial character, and a visual search task. The picture (8°) appeared in the center of the screen for 1,200 ms, followed by a 300-ms blank interval, and the corresponding artificial character (3°) also for 1,200 ms. Participants were then asked to complete a visual search task (Fig. 1B) by finding the target artificial character among six choices: the target, a related distractor, and four unrelated distractors. The related distractor shared the same semantic radical (i.e., semantic-match) or the same control radical (i.e., control-match) with the target, or neither (i.e., mismatch). These related distractors were artificial characters selected from the experimental stimuli. The four unrelated distractors were less complex (i.e., single-character) symbols compared to the artificial characters, and the same across trials. As quickly and accurately as possible, participants determined top or bottom position of the gap on the Landolt square surrounding the target artificial character (  -top,  -bottom). Each picture–artificial-character pair repeated six times with each distractor type condition (i.e., semantic-match, control-match, and mismatch) repeated two times, resulting in 216 learning trials completed in six blocks. Participants were not informed about the relation between the pictures and artificial characters prior to the experiment.

In the testing phase (Fig. 1E), a picture (8°) appeared on screen for 1,200 ms. After a 500-ms blank, participants selected from four options—a target, a semantic-match distractor, a control-match distractor, and a mismatch distractor—the artificial character most likely to follow that picture. The semantic-match distractor combined the correct semantic radical with an incorrect control radical; the control-match distractor combined the correct control radical with an incorrect semantic radical; and these two incorrect radicals formed a mismatch distractor. The incorrect semantic and control radicals were selected from the learning phase stimuli, ensuring that all distractors appeared and were paired with other learning pictures during the learning phase to minimize the familiarity effect. Participants encountered each of the 36 picture–artificial-character pairs once. Together, the learning and testing phases lasted approximately 45 min.

#### Data analytical approach

First, to examine online statistical learning effects and the internal working memory representations of abstract and item-specific information, we analyzed reaction times (RTs) of the visual search task in the learning phase. To trim the data, trials with incorrect visual searches (4.2%) and RTs above or below 2.5 standard deviations (*SD*s) per condition (1.0%) were eliminated from formal analysis. A generalized linear mixed model (GLMM) was then performed, with distractor type (semantic-match, control-match, and mismatch), probability (high, moderate, and low), and their interaction as independent variables. For distractor type, we coded the semantic-match, control-match, and mismatch conditions as 1/3, −2/3, and 1/3 to compare semantic-match with control-match, where the difference in RTs indicated the statistical learning effect and the online prioritization of abstract information, and as 1/3, 1/3, and −2/3 to compare semantic-match with mismatch, with their RT difference indicating the working memory representation of abstract information. We focused on the first comparison. For probability, we coded high, moderate, and low as −1/3, 2/3, and −1/3 to compare moderate with high probability, and as −1/3, −1/3, and 2/3 to compare low with high probability. Following the maximal random effects principle and convergence requirement (Barr et al., 2013; Singmann & Kellen, 2019), we included by-subject and by-item random intercepts. The GLMM model used a gamma family with a log function, which is suggested for positively skewed RTs (Lo & Andrews, 2015).

Second, to explore the offline representations of abstract and item-specific information, recognition responses of the testing phase were analyzed using a two-way repeated-measures analysis of variance (ANOVA), with selected item type (target, semantic-match, control-match, and mismatch) and probability (high, moderate, and low) as independent variables. When Mauchly’s test showed the assumption of sphericity was violated, adjusted *p* values via the Greenhouse–Geisser correction method were reported. For all post hoc comparisons, *p* values were adjusted using the Bonferroni method.

### Results and discussion

**Fig. 2:**
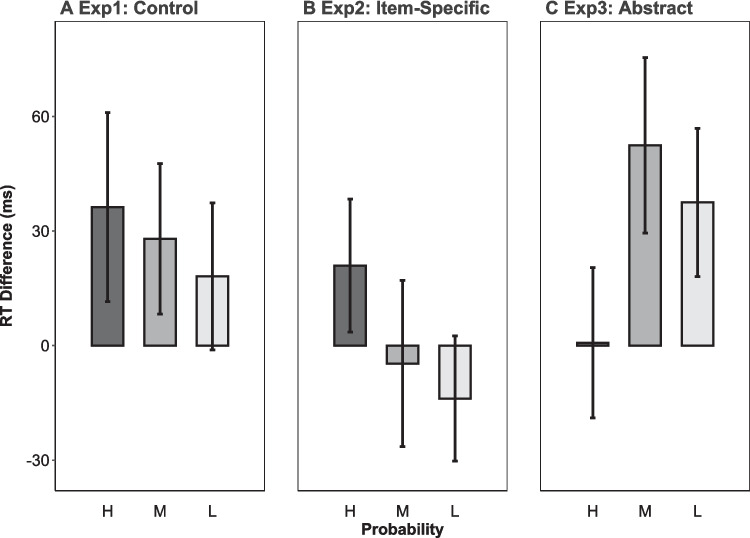
The reaction time (RT) difference between semantic-match and control-match conditions across high (H), moderate (M), and low (L) probability levels in the control (Experiment 1) (**A**), item specific (Experiment 2) (**B**), and abstract encoding (Experiment 3) (**C**) conditions during the visual search phase. Error bars denote standard errors

Figure 2A shows the RT difference between semantic-match and control-match distractor types during the visual search phase across different probability levels. The GLMM results for the visual search task revealed that participants responded slower for the semantic-match compared to the control-match condition (β = 0.013, *SE* = 0.005, *p* = .011), indicating successful statistical learning and an internal prioritization towards abstract over item-specific information. No significant interaction was found between probability and distractor type (*p* > .05). However, the main effect of probability was significant, with longer RT for moderate (β = 0.012, *SE* = 0.005, *p* = .024) but not low (*p* > .05) compared with high probability items.

**Fig. 3:**
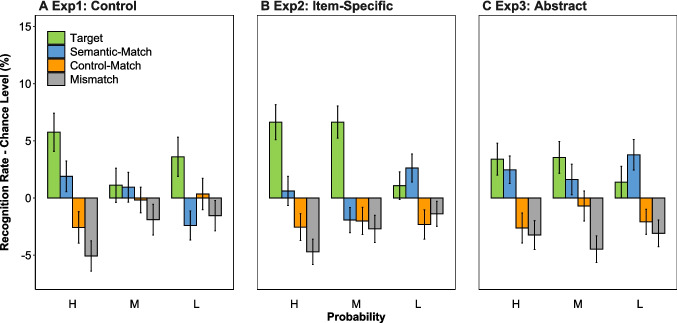
The difference between recognition rates and chance level for selected item types (target, semantic-match, control-match, mismatch) across high (H), moderate (M), and low (L) probability levels in the control (Experiment 1) (**A**), item-specific (Experiment 2) (**B**), and abstract encoding (Experiment 3) (**C**) conditions during the testing phase. Error bars denote standard errors. (Color figure online)

For recognition rates during the testing phase (see details in Supplemental Table S3), illustrated in Fig. 3A, targets were selected significantly above the 25% chance level for high (30.8%), *t*(96) = 3.45, *p* < .001, *d* = 0.35, and low (28.6%), *t*(96) = 2.11, *p* = .037, *d* = 0.21, but not moderate (26.1%, *p* > .05) probability items. A two-way ANOVA on participants’ recognition rates yielded a significant main effect of selected item type, *F*(2.28, 219.32) = 5.71, *p* = .002. Specifically, participants selected more targets (28.5%) compared to mismatch distractors (22.2%), *t*(96) = 3.26, *p* = .009, while other comparisons were not significant (*p*s >.05). The interaction between selected item type and probability, *F*(5.84, 560.42) = 2.68, *p* = .015, was significant. Specifically, participants selected more targets for high (30.8%) compared with moderate probability items (26.1%), *t*(96) = 2.57, *p* = .035, and demonstrated a trend of selecting more semantic-match distractors for high (26.9%) compared with low probability items (22.6%), *t*(96) = 2.23, *p* = .084.

Experiment 1 validated the experimental paradigm and revealed a prioritization towards abstract over item-specific representations regardless of the probability level during statistical learning. However, moderate compared with high probability items showed different online processing speeds and offline recognition rates. This result may be due to the different mechanisms involved (Tong et al., 2023; Zhou et al., 2024). Whereas moderate-probability items rely on probabilistic rules that are processed implicitly and stored in the procedural system, high-probability items are governed by deterministic rules stored in the declarative system (Lee et al., 2022). In our study, moderate items involved less explicit resources, and the working memory representations were weaker. Thus, participants spent a longer time searching among visually similar distractors. Additionally, as evidenced by their slightly better recognition rates for semantic-match distractors in high- compared with low-probability items, high probability items may involve more robust abstract processing than low ones. These results suggest that the mechanisms underlying the online prioritization of abstract information may differ across probability levels. To test this hypothesis, we further investigated how a single type of encoding strategy that induced a specific verbalization of the stimuli influenced participants’ internal representations across probability levels. In particular, we prompted participants to use an item-specific encoding strategy in Experiment 2 and an abstract encoding strategy in Experiment 3.

## Experiment 2

### Method

#### Participants

Participants were 108 university students (*M*_age_ = 23.87 ± 3.25 years; 93 women, 15 men) not involved in Experiment 1.

#### Stimuli and procedure

Stimuli and procedure were the same as in Experiment 1 except that a memory recall task was added after the visual search task during the learning phase. For the memory recall task, participants determined whether a real Chinese word (e.g., 面包(*bread*)) represented a specific meaning of a previously shown picture (e.g.,  ). Half of the trials were foils involving real words with incorrect meanings (e.g., 鼓(*drum)*) randomly selected from the experimental stimuli (Fig. 1D).

#### Data analytical approach

The same data analysis as Experiment 1 was applied. For the online visual search phase, trials with incorrect visual searches or incorrect memory recall (7.9%) and log RTs above or below 2.5 standard deviations per condition (0.9%) were eliminated from formal analysis.

### Results and discussion

As shown in Fig. 2B, unlike Experiment 1, the results for the visual search task revealed that the RT difference between semantic-match and control-match conditions was not significant (*p* > .05), but was marginally moderated by probability, with a larger difference for high compared with low (β = −0.021, *SE* = 0.012, *p* = .092) but not moderate (*p* > .10) probability items. This result suggests that an item-specific encoding strategy hinders the internal prioritization of abstract information especially for low-probability items. However, high-probability items may form robust abstract representations that resist the influence of an item-specific strategy.

For recognition rates during the testing phase, as shown in Fig. 3B, targets for high (31.6%), *t*(107) = 4.33, *p* < .001, *d* = 0.42, and moderate (31.6%), *t*(107) = 4.72, *p* < .001, *d* = 0.45, items, and semantic-match distractors for low-probability items (27.6%), *t*(107) = 2.13, *p* = .035, *d* = 0.21, were selected above the 25% chance level. A two-way ANOVA yielded a significant main effect of selected item type, *F*(2.55, 273.17) = 16.46, *p* < .001. Participants selected more targets (29.8%) than semantic-match (25.4%), control-match (22.7%), and mismatch distractors (22.1%; all *p*s < .01), and more semantic-match than mismatch distractors, *t*(107) = 2.86, *p* = .031. The interaction between selected item type and probability, *F*(5.49, 587.33) = 3.14, *p* = .006, was significant. However, unlike Experiment 1, participants selected more targets for both high (31.6%), *t*(107) = 2.91, *p* = .013, and moderate (31.6%), *t*(107) = 2.89, *p* = .014, compared with low-probability items (26.1%), and fewer semantic-match distractors for moderate (23.1%) compared with low-probability items (27.6%), *t*(107) = 2.82, *p* = .017.

Experiment 2 showed that when participants were implicitly oriented to an item-specific encoding strategy to verbalize the pictures, online representations shifted to less abstract information, especially for low- compared with high-probability items. Furthermore, compared with Experiment 1, participants showed more accurate representations during offline recognition for moderate items. These results supported our hypothesis that an item-specific encoding strategy increased item-specific representations for lower probability items. To further test our hypothesis that an abstract encoding strategy would enhance abstract representations for high-probability items, the encoding strategy was changed to an abstract condition in Experiment 3.

## Experiment 3

### Method

#### Participants

Participants consisted of 108 university students (*M*_age_ = 24.03 ± 4.05 years; 90 women, 18 men) not involved in the previous two experiments.

#### Stimuli and procedure

Stimuli and procedure were the same as in Experiment 2, except that during the memory recall task, participants determined whether a real Chinese semantic category word (e.g., 食物 (*food*)) represented the category of a previously shown picture (e.g.,  ). Half of the trials were foils involving real words with incorrect semantic categories (e.g., 运动 (*sport*)) randomly selected from the experimental stimuli.

#### Data analytical approach

Following the same data analysis as in Experiment 2, trials with incorrect visual searches or incorrect memory recall (9.3%) and log RTs above or below 2.5 standard deviations per condition (0.9%) were eliminated from formal analysis.

Furthermore, to investigate our second research question of how encoding strategies influenced the representations of abstract and item-specific information across probability levels, we tested a full GLMM model, with distractor type (semantic-match, control-match, and mismatch), probability (high, moderate, and low), encoding strategy (Experiment 1: control; Experiment 2: item-specific; and Experiment 3: abstract), and their interactions as independent variables. Distractor type and probability were coded following Experiments 1 and 2. For encoding strategy, we coded control, item specific, and abstract as −1/3, 2/3, and −1/3 to compare item specific with control, and as −1/3, −1/3, and 2/3 to compare abstract with item specific. We included by-subject and by-item random intercepts.

To investigate between-experiment differences of recognition responses of the testing phase, a three-way repeated-measures ANOVA was conducted, with selected item type (target, semantic-match, control-match, and mismatch), probability (high, moderate, and low), and encoding strategy (item specific, abstract, and control) as independent variables.

### Results and discussion

As shown in Fig. 2C, GLMM results for the visual search task in Experiment 3 revealed a longer RT for the semantic-match compared with the control-match conditions (β = 0.018, *SE* = 0.005, *p* < .001), indicating an internal prioritization of abstract over item-specific information. Furthermore, the RT difference between semantic-match and control-match conditions was moderated by probability, with a larger difference for moderate (β = 0.033, *SE* = 0.013, *p* = .009) but not low (*p* = .104) compared with high-probability items. The full GLMM model for between-experiment comparisons revealed a significant three-way interaction among distractor type, probability, and encoding strategy. Specifically, compared with Experiment 2, Experiment 3 revealed a larger effect for the RT difference between semantic-match and control-match conditions when comparing moderate (β = 0.048, *SE* = 0.018, *p* = .007) and low (β = 0.042 *SE* = 0.018, *p* = .017) with high-probability items. These results suggest that an abstract encoding strategy enhances abstract representations particularly for moderate- and low-probability items.

For recognition rates during the testing phase, depicted in Fig. 3C, targets for high (28.4%), *t*(107) = 2.42, *p* = .017, *d* = 0.23, and moderate (28.5%), *t*(107) = 2.56, *p* = .012, *d* = 0.25, items, and semantic-match distractors for high (27.5%), *t*(107) = 2.05, *p* = .042, *d* = 0.20, and low-probability items (28.8%), *t*(107) = 2.83, *p* = .006, *d* = 0.27, were selected above the 25% chance level. A two-way ANOVA yielded a significant main effect of selected item type, *F*(2.77, 295.95) = 12.27, *p* < .001, with participants selecting more targets (27.8%) and semantic-match distractors (27.6%) compared with control-match (23.2%) and mismatch distractors (21.4%; all *p*s < .01). Unlike Experiments 1 and 2, the interaction between selected item type and probability was not significant (*p* > .05). Between-experiment comparisons revealed a significant three-way interaction among selected item type, probability, and encoding strategy, *F*(11.60, 1798.29) = 1.88, *p* = .034, indicating that encoding strategy further influenced the recognition rates of targets for moderate probability items, *F*(2, 310) = 3.70, *p* = .026, and semantic-match distractors for moderate, *F*(2, 310) = 2.33, *p* = .099, and low-probability items, *F*(2, 310) = 6.34, *p* = .002. Specifically, the recognition rate of targets for moderate items was larger for Experiment 2 (31.6%) compared with Experiment 1 (26.1%), *t*(310) = 2.71, *p* = .022. The recognition rate of semantic-match distractors for low-probability items was larger for both Experiment 3 (28.8%), *t*(310) = 3.38, *p* = .002, and Experiment 2 (27.6%), *t*(310) = 2.74, *p* = .019, compared to Experiment 1 (22.6%). These results suggest that an item-specific strategy enhances the overall processing for moderate items, and an abstract encoding strategy enhances the retrieval of abstract information for moderate and low probability items.

## General discussion

Using a novel learning-memory representation paradigm, this study demonstrated that adults implicitly extracted semantic categorical patterns of individual items. They prioritized abstract over item-specific information across varying probability levels within working memory (Experiment 1). However, this prioritization depended on probability levels and encoding strategies: an item-specific strategy hindered abstract prioritization for low-probability items (Experiment 2), whereas an abstract strategy enhanced abstract prioritization for moderate- and low-probability items (Experiment 3). These findings suggest an abstraction-prioritized, probability-variant, and strategy-regulated mechanism that underpins the interplay between online statistical learning and working memory, allowing for flexible organization of memory representations of both abstract and item-specific information.

A key finding is that individuals automatically prioritized abstract over item-specific information, regardless of probability level. Experiment 1 showed that, without a specific encoding strategy, participants spent more time searching semantic-matched distractors than control-matched ones across high-, moderate-, and low-probability levels. This supports previous research showing that individuals can extract high-level statistical information, such as semantic categories (Brady & Oliva, 2008; Emberson & Rubinstein, 2016; Jung et al., 2020). While Emberson and Rubinstein’s (2016) offline tests found that learners recognized category pairs only when item-level relations were absent, other studies suggested that an automatic abstraction process occurred during encoding, linking new inputs to existing memory traces (Zhou et al., 2023). Our results reinforce this latter view by showing that, during online statistical learning, working memory represented abstract semantic categories even when item-level relations were present (i.e., each picture corresponded to a unique artificial character). Moreover, unlike those of previous studies focusing solely on the existence of abstraction, our findings demonstrate a prioritization of abstract over item-specific representations, thus indicating a hierarchical organization where abstract categories supersede item-specific details for flexible memory processing.

Furthermore, Experiments 2 and 3 demonstrated that memory prioritization during statistical learning is jointly influenced by both probability and encoding strategy. In Experiment 2, where participants were prompted to memorize the item-specific meanings, abstract prioritization was absent. This is explained by C-HORSE as the competition between encoding specific items and extracting shared features (Schapiro et al., 2017; Sučević & Schapiro, 2023). An item-specific encoding strategy emphasizes episodic details of individual instances, reducing the tendency to encode abstract information. Notably, internal prioritization did not shift towards item-specific details, likely because abstract encoding is the automatic default, especially for high-probability items with predictable patterns. Supporting this explanation, we observed a trend where abstract information was prioritized for high- but not low-probability items, possibly because deterministic semantic patterns in high-probability items evoked stronger abstract encoding and diminished reliance on item-specific strategy.

Contrary to our initial hypothesis that an abstract encoding condition would enhance the abstraction of high-probability items, Experiment 3 showed stronger representations for moderate- and low-probability items. One explanation is that the abstract encoding condition reinforced learners’ beliefs in the deterministic patterns of high-probability items, leading to reduced prioritization of abstract information that predicts these associations. This aligns with prior research indicating that predictable items elicit expectation suppression, decreasing their processing (e.g., Summerfield & De Lange, 2014), especially when predictions are highly certain (Zhang et al., 2023). Another explanation involves multiple mechanisms for moderate- and low-probability items: An abstract encoding strategy emphasizes the processing of regular items, while simultaneously reducing item-specific processing of irregular items (i.e., prediction errors). Previous research has suggested that probabilistic learning relies on both an abstraction mechanism that detects consistent patterns among regular items (Salet et al., 2021) and an item-specific mechanism that memorizes irregular, nonconforming events to minimize prediction errors (Bein et al., 2021). This explanation is also supported by our GLMM analysis showing that the prioritization of abstract information differed significantly between regular and irregular items (see Supplemental Table S5 and Fig. S1).

Overall, our result that working memory prioritized abstract information for regular but not irregular items in the control condition supports the notion that consistent patterns involve more abstract processing, while prediction errors engage more item-specific processing (Bein et al., 2021; Salet et al., 2021). However, the item-specific encoding strategy hindered the prioritization of abstract information for regular items, while the abstract encoding strategy enhanced the prioritization of abstract information for irregular items. This extends previous research that prediction errors improve item-specific memory (Jang et al., 2019) by demonstrating that prediction error processing can also flexibly prioritize abstract information.

These findings connecting statistical learning and working memory have significant implications for both. The automatic prioritization of abstract information underscores that working memory representations are shaped by statistical regularities. While previous research has shown that working memory selectively encodes sensory inputs due to limited working memory resources (e.g., Fu et al., 2023), most studies have focused on physical attributes of individual objects or features (Bruning & Lewis-Peacock, 2020; Fu et al., 2021; Umemoto et al., 2010). Our study extends this by suggesting that entire objects, such as artificial characters, and their separated parts (e.g., semantic and control radicals), each containing distinct statistical patterns, can also be selectively manipulated and retained in working memory. Moreover, working memory allocated more resources to abstract information, likely because statistical learning conserved limited memory resources by integrating multiple items into generalized abstract categories. Importantly, since participants were never explicitly instructed to memorize picture-artificial character pairs, the representations of these pairs were relatively implicit. Thus, our study represents an experimental investigation into the implicit component of working memory during statistical learning (Arciuli, 2017; Janacsek & Nemeth, 2013).

Our findings also extend the C-HORSE model (Schapiro et al., 2017; Sučević & Schapiro, 2023), which emphasizes the parallel processes that develop both abstract and item-specific representations. We demonstrate that these representations interact with working memory, which assigns prioritized weights to abstract information. To fully capture learning performances across various probabilistic inputs, future models should integrate the working memory mechanisms, especially regarding the online organizations of flexible, structured representations.

Moreover, the influence of probability on working memory engagement varies across levels. High (i.e., deterministic) and moderate and low (i.e., probabilistic) inputs involve distinct operations, reflecting the complex working memory mechanisms underlying statistical learning (Janacsek & Nemeth, 2013; Lee et al., 2024; Zhou et al., 2024). Previous studies have provided mixed views, with some suggesting that high-probability items are learned automatically with minimum attention (e.g., Tong et al., 2023), while others found a positive correlation between working memory capacity and learning these items (e.g., Zhou et al., 2024). Similarly, some propose that moderate- and low-probability items rely on working memory for complex pattern extraction (e.g., Conway, 2020), whereas others suggest implicit learning mechanisms predominate (Lee et al, 2024).

Unlike these previous studies that proposed a fixed mechanism for each probability level, our findings support a flexible, multiple-mechanism account: high-probability (i.e., deterministic) inputs maintain some abstract processing even when abstraction is less necessary in the item-specific encoding condition; however, when abstraction is redundant in the abstract encoding condition, less emphasis is placed on it. For moderate- and low-probability (i.e., probabilistic) inputs, encoding strategy flexibly determined whether detailed or generalized information is processed. Overall, our findings support a flexible, multiple-mechanism account, where working memory functions as an adaptable system that underpins various mechanisms for processing and managing both deterministic (predictable) and probabilistic (uncertain) inputs.

Finally, although this study focused on encoding process in statistical learning, the recognition test results from Experiments 2 and 3 further support the prioritization of abstract representations. Participants showed significantly higher recognition rates for semantic-match distractors than for mismatch or control-match items, indicating that abstract representations are effectively encoded and retained for subsequent memory retrieval processes. These findings open new avenues for future research, such as examining how explicit tasks, like asking participants to categorize information based on learned concepts, influence the prioritization of abstract information. Such studies could clarify how the memory system revises, updates, and reorganizes representations to optimally predict and process varying probabilistic inputs. Ultimately, this will advance current understanding of the mechanisms that support cognitive efficiency and adaptability in complex, dynamic environments.

In conclusion, this study demonstrates that humans naturally tend to abstract probabilistic relations from environmental inputs, prioritizing abstract over item-specific information. This abstraction process is influenced by the encoding strategy: An item-specific strategy emphasizes exemplars, while an abstract strategy enhances the prioritization of abstract information, particularly for moderate- and low- compared with high-probability items. These findings suggest that learning generally defaults to abstracting probabilistic inputs, with multiple mechanisms flexibly adapting based on the context that favored either item-specific or abstract encoding. The presence of multiple mechanisms for processing different probability levels highlights the human mind’s capacity to adapt efficiently to the multifaceted complexities inherent in our environment, demonstrating the versatility of our cognitive systems.

## Supplementary Information

Below is the link to the electronic supplementary material.

Supplementary file1 (DOCX 489 KB)

## Data Availability

The data are available in the Open Science Framework repository (https://osf.io/a6njx/?view_only=bfdcbbc49fb940708ea90dec4f347fc0). The materials are available from the corresponding author on reasonable request.

## Code availability

The R script to reproduce all analyses are available in the Open Science Framework repository (https://osf.io/a6njx/?view_only=bfdcbbc49fb940708ea90dec4f347fc0).
