# Supplementary material for: Statistical learning prioritizes abstract over item-specific representations
Source: Psychon Bull Rev. 2025 Sep 3;32(6):3264–75. doi: 10.3758/s13423-025-02757-8 (PMC12627146; doi:10.3758/s13423-025-02757-8)
Supplement: Supplementary file 1 — Supplementary file1 (DOCX 489 KB) [file 13423_2025_2757_MOESM1_ESM.docx]

Table S1

Generalized Linear Mixed Model Results for Reaction Times of the Visual Search Phase During Learning in the Control (Experiment 1), Item Specific (Experiment 2), and Abstract Encoding (Experiment 3) Conditions

| Variables | β | *SE* | *z* | *p(>\|z\|)* | 95% CI |
| --- | --- | --- | --- | --- | --- |
| **Experiment 1 (Control)** |  |  |  |  |  |
| (intercept) | 7.321 | 0.022 | 331.01 | **<.001***** | [7.277, 7.364] |
| Distractor type (H1) | 0.013 | 0.005 | 2.53 | **0.011*** | [0.003, 0.023] |
| Distractor type (H2) | 0.064 | 0.005 | 12.35 | **<.001***** | [0.054, 0.074] |
| Probability (M) | 0.012 | 0.005 | 2.26 | **0.024*** | [0.002, 0.022] |
| Probability (L) | 0.006 | 0.005 | 1.16 | 0.247 | [-0.004, 0.016] |
| Distractor type (H1) × Probability (M) | -0.002 | 0.013 | -0.12 | 0.903 | [-0.027, 0.024] |
| Distractor type (H2) × Probability (M) | 0.008 | 0.013 | 0.61 | 0.540 | [-0.017, 0.033] |
| Distractor type (H1) × Probability (L) | -0.008 | 0.013 | -0.64 | 0.523 | [-0.033, 0.017] |
| Distractor type (H2) × Probability (L) | -0.012 | 0.013 | -0.93 | 0.355 | [-0.037, 0.013] |
| **Experiment 2 (Item-Specific)** |  |  |  |  |  |
| (intercept) | 7.330 | 0.020 | 363.06 | **<.001***** | [7.291, 7.370] |
| Distractor type (H1) | 0.001 | 0.005 | 0.25 | 0.799 | [-0.009, 0.011] |
| Distractor type (H2) | 0.061 | 0.005 | 12.27 | **<.001***** | [0.051, 0.071] |
| Probability (M) | 0.003 | 0.005 | 0.59 | 0.556 | [-0.007, 0.013] |
| Probability (L) | 0.006 | 0.005 | 1.14 | 0.253 | [-0.004, 0.015] |
| Distractor type (H1) × Probability (M) | -0.014 | 0.012 | -0.17 | 0.244 | [-0.038, 0.010] |
| Distractor type (H2) × Probability (M) | 0.004 | 0.012 | 0.30 | 0.760 | [-0.020, 0.027] |
| Distractor type (H1) × Probability (L) | -0.021 | 0.012 | -1.68 | **0.092^†^** | [-0.045, 0.003] |
| Distractor type (H2) × Probability (L) | -0.016 | 0.012 | -1.36 | 0.175 | [-0.040, 0.007] |
| **Experiment 3 (Abstract)** |  |  |  |  |  |
| (intercept) | 7.318 | 0.021 | 354.87 | **<.001***** | [7.278, 7.359] |
| Distractor type (H1) | 0.018 | 0.005 | 3.42 | **<.001***** | [0.008, 0.028] |
| Distractor type (H2) | 0.059 | 0.005 | 11.56 | **<.001***** | [0.049, 0.070] |
| Probability (M) | 0.009 | 0.005 | 1.84 | **0.065^†^** | [-0.001, 0.020] |
| Probability (L) | -0.003 | 0.005 | 0.52 | 0.600 | [-0.013, 0.007] |
| Distractor type (H1) × Probability (M) | 0.033 | 0.013 | 2.60 | **0.009**** | [0.008, 0.058] |
| Distractor type (H2) × Probability (M) | 0.045 | 0.013 | 3.55 | **<.001***** | [0.020, 0.069] |
| Distractor type (H1) × Probability (L) | 0.021 | 0.013 | 1.63 | 0.104 | [-0.004, 0.046] |
| Distractor type (H2) × Probability (L) | 0.002 | 0.013 | 0.19 | 0.847 | [-0.022, 0.027] |

*Note.* Hypothesis 1 and 2 (H1 and H2) for distractor type were to compare semantic-match with control-match and mismatch conditions, respectively. *** *p* < .001; ** *p* < .01; * *p* < .05; **^†^** *p* < .10.

Table S2

Generalized Linear Mixed Model Results for Reaction Times of the Visual Search Phase During Learning

| Variables | β | *SE* | *z* | *p(>\|z\|)* | 95% CI |
| --- | --- | --- | --- | --- | --- |
| (intercept) | 7.323 | 0.012 | 607.64 | **<.001***** | [7.300, 7.347] |
| Distractor type (H1) | 0.011 | 0.003 | 3.66 | **<.001***** | [0.005, 0.017] |
| Distractor type (H2) | 0.062 | 0.003 | 20.88 | **<.001***** | [0.056, 0.067] |
| Probability (M) | 0.008 | 0.003 | 2.73 | **0.006**** | [0.002, 0.014] |
| Probability (L) | 0.003 | 0.003 | 1.03 | 0.302 | [-0.003, 0.009] |
| Encoding (H1) | -0.010 | 0.029 | -0.33 | 0.744 | [-0.067, 0.048] |
| Encoding (H2) | -0.012 | 0.028 | -0.43 | 0.670 | [-0.068, 0.044] |
| Distractor type (H1) × Probability (M) | 0.005 | 0.007 | 0.75 | 0.451 | [-0.009, 0.020] |
| Distractor type (H2) × Probability (M) | 0.019 | 0.007 | 2.63 | **0.009**** | [0.005, 0.033] |
| Distractor type (H1) × Probability (L) | -0.003 | 0.007 | -0.35 | 0.723 | [-0.017, 0.012] |
| Distractor type (H2) × Probability (L) | -0.008 | 0.007 | -1.16 | 0.244 | [-0.023, 0.006] |
| Distractor type (H1) × Encoding (H1) | 0.012 | 0.007 | 1.63 | 0.104 | [-0.002, 0.026] |
| Distractor type (H2) × Encoding (H1) | 0.003 | 0.007 | 0.44 | 0.657 | [-0.011, 0.017] |
| Distractor type (H1) × Encoding (H2) | 0.017 | 0.007 | 2.29 | **0.022*** | [0.002, 0.031] |
| Distractor type (H2) × Encoding (H2) | -0.001 | 0.007 | -0.19 | 0.852 | [-0.015, 0.013] |
| Probability (M) × Encoding (H1) | 0.009 | 0.007 | 1.20 | 0.228 | [-0.005, 0.023] |
| Probability (L) × Encoding (H1) | 0.000 | 0.007 | 0.05 | 0.960 | [-0.014, 0.015] |
| Probability (M) × Encoding (H2) | 0.006 | 0.007 | 0.90 | 0.367 | [-0.008, 0.020] |
| Probability (L) × Encoding (H2) | -0.008 | 0.007 | -1.17 | 0.240 | [-0.022, 0.006] |
| Distractor type (H1) × Probability (M) × Encoding (H1) | 0.014 | 0.018 | 0.76 | 0.447 | [-0.021, 0.049] |
| Distractor type (H2) × Probability (M) × Encoding (H1) | 0.005 | 0.018 | 0.27 | 0.789 | [-0.030, 0.039] |
| Distractor type (H1) × Probability (L) × Encoding (H1) | 0.013 | 0.018 | 0.72 | 0.470 | [-0.022, 0.048] |
| Distractor type (H2) × Probability (L) × Encoding (H1) | 0.005 | 0.018 | 0.27 | 0.786 | [-0.030, 0.040] |
| Distractor type (H1) × Probability (M) × Encoding (H2) | 0.048 | 0.018 | 2.72 | **0.007**** | [0.013, 0.082] |
| Distractor type (H2) × Probability (M) × Encoding (H2) | 0.041 | 0.017 | 2.38 | **0.017*** | [0.007, 0.076] |
| Distractor type (H1) × Probability (L) × Encoding (H2) | 0.042 | 0.018 | 2.39 | **0.017*** | [0.008, 0.077] |
| Distractor type (H2) × Probability (L) × Encoding (H2) | 0.019 | 0.017 | 1.11 | 0.268 | [-0.015, 0.054] |

*Note.* Hypothesis 1 and 2 (H1 and H2) for distractor type were to compare semantic-match and control-match with mismatch conditions, respectively. Hypothesis 1 and 2 (H1 and H2) for encoding strategy (Encoding) were to compare item-specific with control conditions, and abstract with item-specific conditions, respectively. *** *p* < .001; ** *p* < .01; * *p* < .05.

Table S3

Mean Recognition Rates of Selected Item Types Across High (H), Moderate (M), and Low (L) Probability Levels in the Control (Experiment 1), Item Specific (Experiment 2), and Abstract Encoding (Experiment 3) Conditions During the Testing phase

| Experiment | Probability | Selected Item Type | Mean Recognition Rates (SD) | *t* | *p (>\|t\|)* | *d* | *BF_10_* |
| --- | --- | --- | --- | --- | --- | --- | --- |
| Experiment 1: Control (*n* = 97) | H | target | 30.8 (16.4) | 3.45 | **<.001***** | 0.35 | 26.70 |
|  |  | semantic match | 26.9 (13.2) | 1.41 | .162 | 0.14 | 0.29 |
|  |  | control match | 22.4 (13.6) | -1.87 | **.065^†^** | -0.19 | 0.60 |
|  |  | mismatch | 19.9 (13.0) | -3.83 | **<.001***** | -0.39 | 86.12 |
|  | M | target | 26.1 (14.7) | 0.75 | .457 | 0.08 | 0.15 |
|  |  | semantic match | 26.0 (12.8) | 0.73 | .470 | 0.07 | 0.14 |
|  |  | control match | 24.8 (11.1) | -0.15 | .879 | -0.02 | 0.11 |
|  |  | mismatch | 23.1 (13.2) | -1.41 | .162 | -0.14 | 0.29 |
|  | L | target | 28.6 (16.8) | 2.11 | **.037*** | 0.21 | 0.93 |
|  |  | semantic match | 22.6 (12.6) | -1.89 | **.062^†^** | -0.19 | 0.62 |
|  |  | control match | 25.3 (13.5) | 0.25 | .803 | 0.03 | 0.12 |
|  |  | mismatch | 23.5 (13.1) | -1.16 | .247 | -0.12 | 0.22 |
| Experiment 2: Item-Specific (*n* = 108) | H | target | 31.6 (15.9) | 4.33 | **<.001***** | 0.42 | 485.54 |
|  |  | semantic match | 25.6 (13.3) | 0.48 | .630 | 0.05 | 0.12 |
|  |  | control match | 22.5 (12.2) | -2.16 | **.033*** | -0.21 | 0.99 |
|  |  | mismatch | 20.3 (11.5) | -4.27 | **<.001***** | -0.41 | 391.73 |
|  | M | target | 31.6 (14.6) | 4.72 | **<.001***** | 0.45 | 2062.20 |
|  |  | semantic match | 23.1 (11.4) | -1.75 | **.082^†^** | -0.17 | 0.47 |
|  |  | control match | 23.0 (12.3) | -1.69 | **.093^†^** | -0.16 | 0.42 |
|  |  | mismatch | 22.3 (12.4) | -2.27 | **.025*** | -0.22 | 1.24 |
|  | L | target | 26.1 (12.6) | 0.89 | .376 | 0.09 | 0.16 |
|  |  | semantic match | 27.6 (12.8) | 2.13 | **.035*** | 0.21 | 0.94 |
|  |  | control match | 22.7 (13.3) | -1.81 | **.074^†^** | -0.17 | 0.51 |
|  |  | mismatch | 23.6 (11.4) | -1.27 | .207 | -0.12 | 0.23 |
| Experiment 3: Abstract (*n* = 108) | H | target | 28.4 (14.6) | 2.42 | **.017*** | 0.23 | 1.71 |
|  |  | semantic match | 27.5 (12.5) | 2.05 | **.042*** | 0.20 | 0.80 |
|  |  | control match | 22.4 (13.7) | -2.00 | **.048*** | -0.19 | 0.72 |
|  |  | mismatch | 21.8 (13.1) | -2.57 | **.011*** | -0.25 | 2.46 |
|  | M | target | 28.6 (14.4) | 2.56 | **.012*** | 0.25 | 2.36 |
|  |  | semantic match | 26.6 (14.0) | 1.20 | .231 | 0.12 | 0.22 |
|  |  | control match | 24.3 (13.6) | -0.53 | .598 | -0.05 | 0.12 |
|  |  | mismatch | 20.5 (12.2) | -3.82 | **<.001***** | -0.37 | 83.75 |
|  | L | target | 26.4 (14.3) | 1.01 | .315 | 0.10 | 0.17 |
|  |  | semantic match | 28.8 (13.9) | 2.83 | **.006**** | 0.27 | 4.63 |
|  |  | control match | 22.9 (11.3) | -1.91 | **.059^†^** | -0.18 | 0.61 |
|  |  | mismatch | 21.9 (12.1) | -2.64 | **.009**** | -0.25 | 2.89 |

*Note.* *** *p* < .001; ** *p* < .01; * *p* < .05; **^†^** *p* < .10.

Table S4

ANOVA Results for the Effects of Probability and Selected Item Type on Recognition Rates in the Control (Experiment 1), Item Specific (Experiment 2), and Abstract Encoding (Experiment 3) Conditions During the Testing Phase

| Experiment | Variables | MS | MSE | df1 | df2 | *F* | *p* | η^2^ |
| --- | --- | --- | --- | --- | --- | --- | --- | --- |
| Experiment 1: Control (*n* = 97) | Probability | 0.00 | 0.00 | 2.00 | 192.00 | 0.00 | 1.000 | .00 |
|  | Selected Item | 2663.07 | 466.22 | 2.28 | 219.32 | 5.71 | **.002**** | .06 |
|  | Probability × Selected Item | 554.57 | 206.61 | 5.84 | 560.42 | 2.68 | **.015*** | .03 |
| Experiment 2: Item-Specific (*n* = 108) | Probability | 0.00 | 0.00 | 2.00 | 214.00 | 0.00 | 1.000 | .00 |
|  | Selected Item | 4684.84 | 284.67 | 2.55 | 273.17 | 16.46 | **<.001***** | .13 |
|  | Probability × Selected Item | 722.45 | 230.19 | 5.49 | 587.33 | 3.14 | **.006**** | .03 |
| Experiment 3: Abstract (*n* = 108) | Probability | 0.00 | 0.00 | 2.00 | 214.00 | 0.00 | 1.000 | .00 |
|  | Selected Item | 3608.68 | 294.16 | 2.77 | 295.95 | 12.27 | **<.001***** | .10 |
|  | Probability × Selected Item | 161.51 | 235.25 | 5.63 | 602.08 | 0.69 | .651 | .01 |

Table S5

Generalized Linear Mixed Model Results for Reaction Times of Visual Search Phase During Learning

| Variables | β | *SE* | *z* | *p(>\|z\|)* | 95% CI |
| --- | --- | --- | --- | --- | --- |
| (intercept) | 7.324 | 0.012 | 593.29 | **<.001***** | [7.300, 7.349] |
| Distractor type (H1) | 0.012 | 0.004 | 3.15 | **<.002**** | [0.004, 0.019] |
| Distractor type (H2) | 0.063 | 0.004 | 17.49 | **<.001***** | [0.056, 0.070] |
| Regularity | -0.005 | 0.003 | -1.84 | **0.066^†^** | [-0.011, 0.000] |
| Encoding (H1) | -0.008 | 0.030 | -0.25 | 0.799 | [-0.067, 0.051] |
| Encoding (H2) | -0.012 | 0.029 | -0.43 | 0.669 | [-0.069, 0.045] |
| Distractor type (H1) × Regularity | -0.010 | 0.007 | -1.31 | 0.190 | [-0.024, -0.005] |
| Distractor type (H2) × Regularity | -0.016 | 0.007 | -2.26 | **0.024*** | [-0.031, -0.002] |
| Distractor type (H1) × Encoding (H1) | 0.016 | 0.009 | 1.83 | **0.068^†^** | [-0.001, 0.034] |
| Distractor type (H2) × Encoding (H1) | 0.005 | 0.009 | 0.58 | 0.559 | [-0.012, 0.023] |
| Distractor type (H1) × Encoding (H2) | 0.031 | 0.009 | 3.53 | **<.001***** | [0.014, 0.049] |
| Distractor type (H2) × Encoding (H2) | 0.009 | 0.009 | 1.01 | 0.310 | [-0.008, 0.026] |
| Regularity × Encoding (H1) | 0.009 | 0.007 | 1.18 | 0.240 | [-0.006, 0.023] |
| Regularity × Encoding (H2) | 0.003 | 0.007 | 0.38 | 0.706 | [-0.011, 0.017] |
| Distractor type (H1) × Regularity × Encoding (H1) | -0.042 | 0.018 | -2.31 | **0.021*** | [-0.077, -0.006] |
| Distractor type (H2) × Regularity × Encoding (H1) | -0.008 | 0.018 | -0.46 | 0.646 | [-0.043, 0.027] |
| Distractor type (H1) × Regularity × Encoding (H2) | -0.039 | 0.018 | -2.21 | **0.027*** | [-0.074, -0.004] |
| Distractor type (H2) × Regularity × Encoding (H2) | -0.035 | 0.018 | -1.99 | **0.046*** | [-0.069, -0.001] |

*Note.* The data excluded the high probability trials because they only had regular but not irregular items. Hypothesis 1 and 2 (H1 and H2) for distractor type were to compare semantic-match with control-match and mismatch conditions, respectively. Hypothesis 1 and 2 (H1 and H2) for encoding strategy (Encoding) were to compare item-specific with control, and abstract with item-specific conditions, respectively. *** *p* < .001; ** *p* < .01; * *p* < .05; **^†^** *p* < .10.

Table S6

Semantic Categories, Part of Speech, and Concreteness for Each Word Stimulus

| Chinese word (English translation) | Semantic category (English translation) | Part of speech | Concreteness |
| --- | --- | --- | --- |
| 面包 (Bread) | 食物 (Food) | Noun | Concrete |
| 拉面 (Noodle) | 食物 (Food) | Noun | Concrete |
| 白饭 (Rice) | 食物 (Food) | Noun | Concrete |
| 沙拉 (Salad) | 食物 (Food) | Noun | Concrete |
| 披萨 (Pizza) | 食物 (Food) | Noun | Concrete |
| 蛋糕 (Cake) | 食物 (Food) | Noun | Concrete |
| 体操 (Gymnastics) | 运动 (Sport) | Noun | Concrete |
| 击剑 (Fencing) | 运动 (Sport) | Verb | Concrete |
| 滑冰 (Ice Skating) | 运动 (Sport) | Verb | Concrete |
| 跳高 (Jumping) | 运动 (Sport) | Verb | Concrete |
| 跑步 (Running) | 运动 (Sport) | Verb | Concrete |
| 游泳 (Swimming) | 运动 (Sport) | Verb | Concrete |
| 床 (Bed) | 家具 (Furniture) | Noun | Concrete |
| 桌子 (Table) | 家具 (Furniture) | Noun | Concrete |
| 衣柜 (Wardrobe) | 家具 (Furniture) | Noun | Concrete |
| 椅子 (Chair) | 家具 (Furniture) | Noun | Concrete |
| 教师 (Teacher) | 职业 (Occupation) | Noun | Concrete |
| 画家 (Painter) | 职业 (Occupation) | Noun | Concrete |
| 消防员 (Firefighter) | 职业 (Occupation) | Noun | Concrete |
| 医生 (Doctor) | 职业 (Occupation) | Noun | Concrete |
| 胶水 (Glue) | 文具 (Stationery) | Noun | Concrete |
| 直尺 (Ruler) | 文具 (Stationery) | Noun | Concrete |
| 巴士 (Bus) | 交通工具 (Transportation) | Noun | Concrete |
| 火车 (Train) | 交通工具 (Transportation) | Noun | Concrete |
| 秋天 (Autumn) | 季节 (Season) | Noun | Abstract |
| 惊讶 (Astonished) | 表情 (Emotion) | Adjective | Abstract |
| 黄金 (Gold) | 金属 (Metal) | Noun | Concrete |
| 鼓 (Drum) | 乐器 (Instrument) | Noun | Concrete |
| 七巧板 (Tangram) | 玩具 (Toy) | Noun | Concrete |
| 鹦鹉 (Parrot) | 鸟类 (Bird) | Noun | Concrete |
| 数字 (Digits) | 数学 (Math) | Noun | Concrete |
| 编织 (Knitting) | 动作 (Action) | Verb | Concrete |
| 沙漠 (Desert) | 自然景观 (Nature) | Noun | Concrete |
| 虚拟现实 (Virtual Reality) | 科技 (Technology) | Noun | Abstract |
| 郁金香 (Tulip) | 花 (Flower) | Noun | Concrete |
| 高塔 (Tower) | 建筑 (Architecture) | Noun | Concrete |

Figure S1

Reaction Time (RT) Differences Between Semantic-Match and Control-Match Conditions Across Regular (Reg) and Irregular (Irreg) Items in the Control (Experiment 1), Item Specific (Experiment 2), and Abstract Encoding (Experiment 3) Conditions During the Visual Search Phase


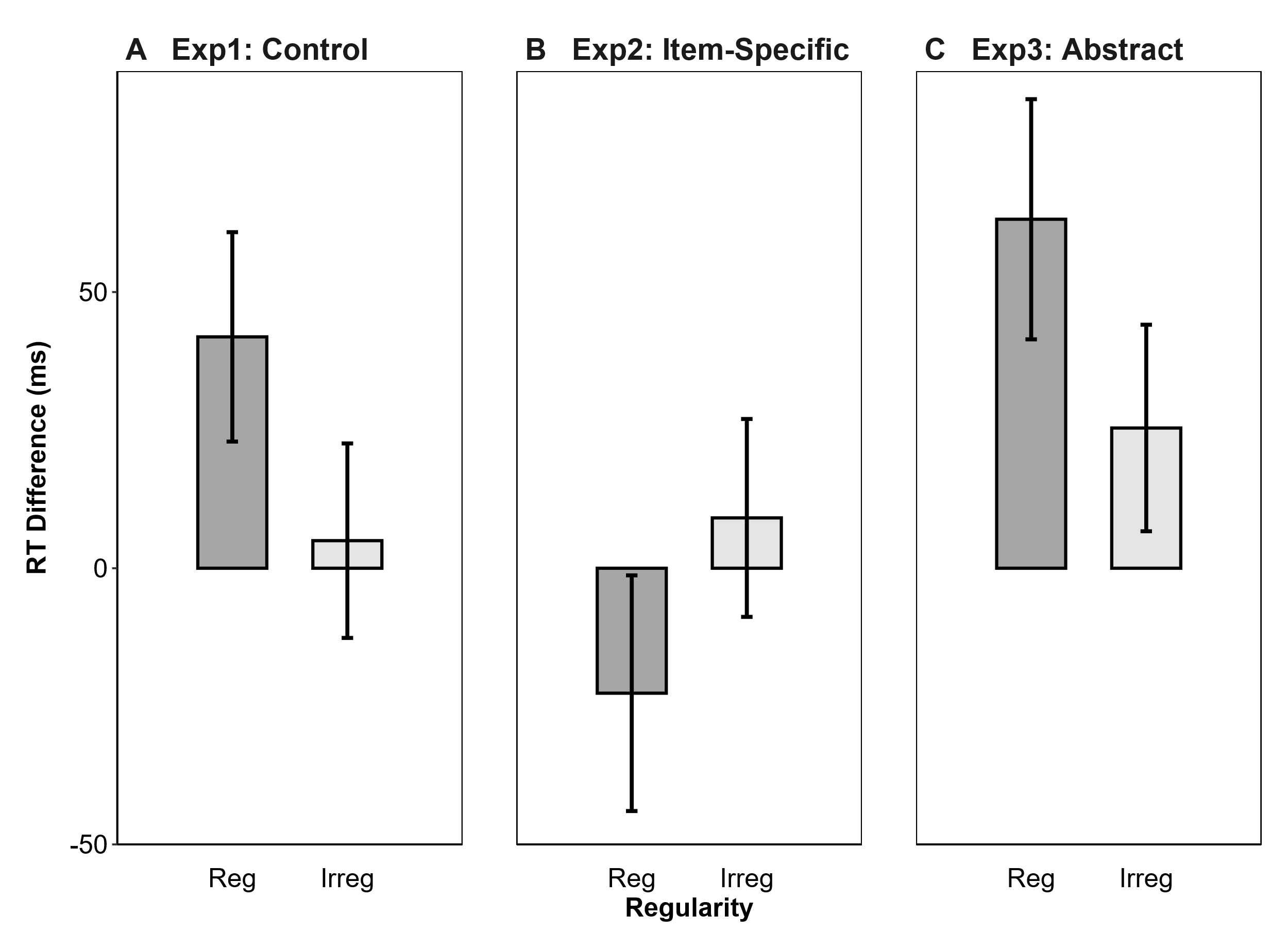


*Note.* Error bars denote standard errors.

*Figure S2*

*Evaluation Scores of the Semantic Similarity (Relatedness) Across Words*


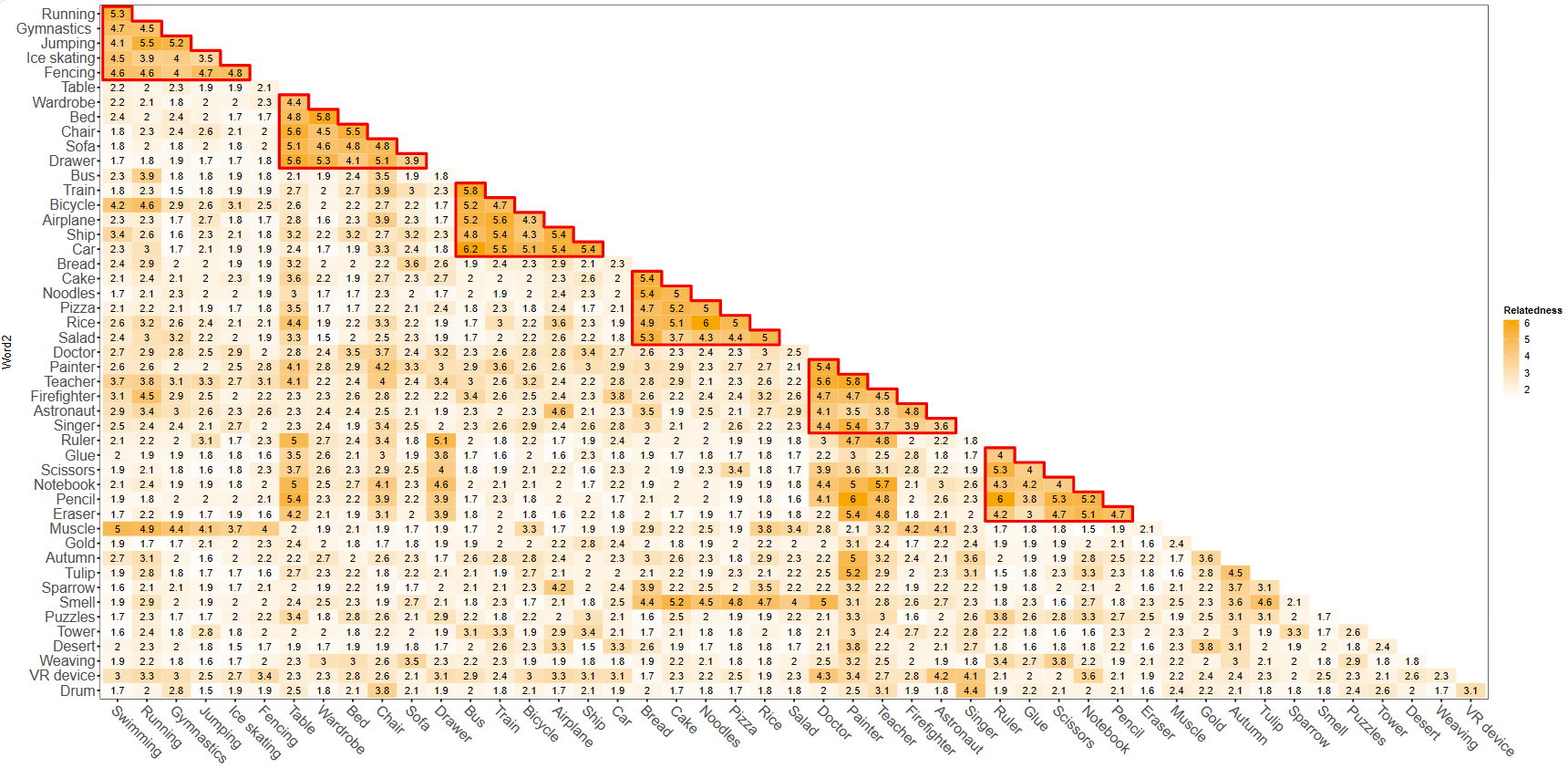


*Note.* The final stimuli comprised 36 words among the 48 words presented here. The evaluation was conducted using the Chinese counterparts of these English words.
